# Cancer morbidity and mortality in individuals with obsessive-compulsive disorder: a population-based and sibling cohort study in Sweden and Denmark

**DOI:** 10.1186/s12916-026-05386-7

**Published:** 2026-11-10

**Authors:** Ioannis Psychogios, Kejia Hu, Joeri Meijsen, Viktor H. Ahlqvist, Jacob Bergstedt, Richard Zetterberg, Brian K. Lee, David Mataix-Cols, Lorena Fernández de la Cruz, Unnur Valdimarsdóttir, Weimin Ye, Mette Nørgaard, Alfonso Buil, Fang Fang

**Affiliations:** 1 Unit of Integrative Epidemiology, Institute of Environmental Medicine, Karolinska Institutet, Stockholm, Sweden; 2 Sleep Medicine Center, Mental Health Center, Sleep Research Laboratory, West China Hospital, National Center for Mental Disorders, Sichuan University, Chengdu, China; 3 West China Biomedical Big Data Center, West China Hospital, Sichuan University, Chengdu, China; 4 Institute of Biological Psychiatry, Amager & Hvidovre Hospital, Mental Health Center Sct. Hans, Copenhagen University Hospital, Roskilde, Denmark; 5 Department of Biomedicine, Aarhus University, Aarhus, Denmark; 6 A.J. Drexel Autism Institute, Drexel University, Philadelphia, PA USA; 7 Department of Epidemiology and Biostatistics, Dornsife School of Public Health, Drexel University, Philadelphia, PA USA; 8 Department of Biostatistics, Epidemiology, and Informatics Perelman School of Medicine, University of Pennsylvania, Philadelphia, PA USA; 9 Centre for Psychiatry Research, Department of Clinical Neuroscience, Karolinska Institutet & Stockholm Health Care Services, Region Stockholm, Stockholm, Sweden; 10 Center of Public Health Sciences, Faculty of Medicine, University of Iceland, Reykjavík, Iceland; 11 Department of Epidemiology, Harvard T. H. Chan School of Public Health, Boston, MA USA; 12 Department of Medical Epidemiology and Biostatistics, Karolinska Institutet, Stockholm, Sweden; 13 Department of Clinical Epidemiology, Aarhus University Hospital, Aarhus, Denmark; 14 Department of Clinical Medicine, Aarhus University, Aarhus, Denmark

**Keywords:** Obsessive-compulsive disorder, Cancer, Morbidity, Mortality, Register-based study

## Abstract

**Background:**

Assessing the risk of cancer morbidity and mortality in individuals with obsessive-compulsive disorder (OCD) is important to identify potential disparities in cancer incidence and prognosis. In this study, we aimed to assess the overall and site-specific cancer risk among individuals with OCD, as well as their cancer-specific and all-cause mortality following a cancer diagnosis.

**Methods:**

This cohort study used population-based matched cohorts and sibling-matched cohorts from Sweden (people born between 1932 and 2010) and Denmark (1932–2009). OCD diagnoses were derived by the Swedish National Patient Register and the Danish Psychiatric Central Register. Each individual with OCD was matched with 20 individuals without OCD on sex, birth year, and calendar time and, in separate analyses, with their OCD-free full siblings. Follow-up began at the index date (OCD diagnosis or matching date) and continued until an incident cancer diagnosis, death, emigration, or end of follow-up (December 31, 2016, in Sweden, or 2015, in Denmark). To assess mortality risk in the Swedish population-matched cohort, individuals diagnosed with cancer were subsequently followed from cancer diagnosis until death, emigration, or end of follow-up.

**Results:**

In the population-based matched cohorts, 1,372 (2.4%) of 57,239 individuals with OCD and 30,376 (2.7%) of 1,144,780 unaffected individuals developed cancer. OCD was not associated with overall cancer risk (pooled HR 0.99, 95% CI 0.94–1.04). In Sweden, OCD was associated with a lower risk of prostate cancer (HR 0.79, 95% CI 0.65–0.97) and melanoma (HR 0.67, 95% CI 0.51–0.89). Following a cancer diagnosis, individuals with OCD showed no clear difference in cancer-specific mortality (HR 1.10, 95% CI 0.97–1.25), but showed higher all-cause mortality (HR 1.27, 95% CI 1.14–1.42), compared with individuals without OCD.

**Conclusions:**

OCD was not associated with overall cancer risk or cancer-specific mortality. However, it was associated with a higher risk of all-cause mortality after cancer diagnosis. There does not seem to be a higher-than-expected cancer burden in this population. However, the higher all-cause mortality after a cancer diagnosis suggests that clinical attention should be directed toward managing comorbid conditions to bridge the mortality gap.

**Supplementary Information:**

The online version contains supplementary material available at https://doi.org/10.1186/s12916-026-05386-7.

## Background

Obsessive-compulsive disorder (OCD) is a psychiatric disorder that affects approximately 1–2% of the global population [1, 2]. It is characterized by the presence of persistent and recurrent obsessions and compulsions that cause marked distress and interfere with daily life [3]. OCD commonly onsets in adolescence and young adulthood [4], and it is often comorbid with other psychiatric disorders such as major depressive disorder and anxiety disorders [4, 5].

Individuals with OCD often face socioeconomic adversities, such as lower educational attainment [6], reduced employment opportunities [7], and impaired interpersonal relationships [8]. In more severe cases, they may neglect personal hygiene, adopt unhealthy lifestyles (e.g., smoking, poor diet, sedentarism) or engage in alcohol and drug misuse [8–10]. These factors can place individuals with OCD at a higher risk of developing various somatic diseases [3].

Cancer remains one of the leading causes of death and morbidity worldwide [11]. Previous research has shown that individuals with psychiatric disorders, such as major depressive disorder and anxiety disorders, may face a higher cancer risk, compared with individuals without these conditions [12, 13]. However, whether this association extends to individuals with OCD remains understudied and sparse. Beyond the aforementioned socioeconomic and lifestyle factors, individuals with OCD frequently experience chronic psychological stress, which can trigger immune dysregulation and chronic inflammation [14]. Furthermore, barriers to healthcare access, including diagnostic delays and lower rates of routine cancer screening, may hinder early detection and intervention [15]. These factors, individually or synergistically, could elevate cancer risk, particularly for malignancies heavily influenced by lifestyle and environmental exposures. Previously, a Taiwanese register-based study reported no increased incidence of cancer compared to the general population [16], while in our prior study using Swedish register data, individuals with OCD, followed from the time of OCD diagnosis, had higher overall mortality but a lower risk of death from neoplasms compared with OCD-free individuals [17]. This association, however, was not observed in sibling-matched analyses.

To clarify the link between OCD, cancer morbidity, and mortality, we analyzed the risk of overall as well as site-specific cancers among individuals with OCD in two large population-based matched cohorts in Sweden and Denmark; we carried out a sibling analysis to account for potential familial confounding; and we performed an analysis to assess the link between OCD and mortality risk following a cancer diagnosis.

## Methods

### Data sources

In both countries, each resident has a unique personal identification number which enables cross-linkage of individual-level data across population and health registers. Demographic variables, familial links, and migration data were retrieved from the Swedish Total Population Register [18] and the Danish Civil Registration System [19], inpatient and outpatient care for psychiatric disorders from the Swedish National Patient Register [20] and the Danish Psychiatric Central Register [21], and cancer diagnoses from the Swedish and Danish Cancer Registers [22, 23]. Data on mortality were derived from the national Cause of Death Registers [24, 25]. In Sweden, we used the longitudinal integrated database for health insurance and labor market studies (LISA) to retrieve information on family income and highest education level [26]. Both countries have similarly structured registers with slight differences in the years of coverage. A detailed overview can be found in ‘Additional file1: Table S1’. This study is reported following the STROBE reporting guidelines for cohort studies (Additional file 1: Table S2).

### OCD diagnoses

We identified OCD diagnoses using inpatient and specialist outpatient records (i.e., psychiatric care delivered by specialized physicians and clinical teams in an outpatient setting), based on country-specific ICD codes (ICD-8: 300.3, ICD-9: 300D, and ICD-10: F42 for Sweden; and ICD-8: 300.39 and ICD-10: F42 for Denmark). Individuals were included if they had at least one recorded OCD diagnosis between January 1st, 1973, and December 31st, 2016, in Sweden, or between January 1st, 1969, and December 31st, 2015, in Denmark. To minimize the risk of misclassification, and following previous research, only individuals diagnosed after the age of 6 years were included [17, 27]. The ICD codes used to identify OCD have been validated in both countries, demonstrating a high positive predictive value [28, 29].

### Cancer diagnosis and cancer-specific mortality

The primary outcome was an incident diagnosis of cancer, identified through the nationwide cancer registers, which have demonstrated high sensitivity and specificity [22, 23]. We restricted the study population to primary, invasive malignant cancer cases identified exclusively from the National Cancer Registries. Non-malignant, in situ, and lesions of uncertain behavior were excluded using anatomical, morphological, and histopathological criteria. In Sweden, malignancies were identified using ICD-7 codes (140–207, including malignant hematological cancers under 200–207) alongside Pathological-Anatomical Diagnosis (PAD) codes. In Denmark, cases were identified using ICD-7 codes (140–207) through 1977, ICD-7 codes mapped to ICD-10 through 2003, and direct ICD-10 coding from 2004 onward. The Danish ICD-10 criteria include ‘C’ codes (C00–C97) as well as select malignant hematological cancers classified under ‘D’ codes (e.g., D45–D47). Considering that risk may vary across different cancer types, we also studied several site-specific cancers. A full list, along with the ICD codes used, is provided in ‘Additional file 1: Table S3’. The secondary outcomes (only explored in the Swedish cohort as the Danish cohort provided an insufficient sample size and low event rates) were cancer-specific and all-cause mortality following a cancer diagnosis, identified through ICD codes (ICD-8 and ICD-9: 140–239 and ICD-10: C00-C99). Cancer-specific mortality was defined as death following a cancer diagnosis where the underlying cause was recorded as any type of cancer according to the Cause of Death Register.

### Covariates

In both countries, we extracted information on parental history of mental illness (ICD-8: 290–315; ICD-9: 290–319; ICD-10: F00-F99) or cancer on the cohort entry date of each participant, in addition to the matching variables of sex and birth year. Parental history of mental illness and cancer was adjusted for to control for potential confounding due to shared genetic liability and early-life environmental stressors, which could influence both the risk of psychiatric disorders and long-term somatic diseases like cancer. For Sweden-specific sensitivity analyses, we collected information on history of mental illness of the index persons other than OCD (ICD-8: 290–315; ICD-9: 290–319; ICD-10: F00-F99), family income, and parental highest education level. For the mortality analysis, we extracted information on the age at cancer diagnosis, as well as the type of cancer.

### Population-based matched cohorts

We used a matched cohort design to assess the risk of cancer among individuals with OCD, compared with those without. The target populations included all individuals born in Sweden during 1932–2010 and in Denmark during 1932–2009.

People with OCD were identified through inpatient and outpatient records, requiring at least one diagnosis after the age of 6. The date of the first recorded OCD diagnosis was defined as their cohort entry date. To focus on incident cancer, we excluded individuals with a prior cancer diagnosis. Each individual with OCD was then randomly matched with 20 individuals with the same sex and birth year, and were free of OCD by the cohort entry date of their matched counterpart [30]. Undiagnosed peers were assigned the same cohort entry date as their matched OCD counterpart (i.e., matched on calendar time). To be eligible for matching, undiagnosed peers had to be residing in Sweden at some point between 1973 and 2016 or between 1969 and 2015 in Denmark, be at least 6 years old, and have no history of cancer as of the cohort entry date of their matched OCD counterpart. All individuals were followed from the cohort entry date until a cancer diagnosis, death, emigration or the end of follow-up (31 December 2016, for Sweden, and 31 December 2015, for Denmark), whichever came first. Undiagnosed peers were also censored if they received a diagnosis of OCD during follow-up, after which they instead had their own undiagnosed peers sampled as controls and followed forward.

Finally, to assess the cancer-specific and overall mortality following a cancer diagnosis, we followed all cancer patients, identified during the follow-up in the Swedish population-based matched cohort, from the date of cancer diagnosis until date of death, emigration, or end of follow-up, whichever came first.

### Sibling-matched cohorts

We linked individuals with OCD to their full siblings who were living in the same country, were at least 6 years old, and had no history of OCD or cancer at the cohort entry date of their exposed sibling. If more than one person per sibling group had an OCD diagnosis, we randomly selected one and compared them with all the siblings that did not have a diagnosis. All siblings were followed from the cohort entry date until the first occurrence of cancer diagnosis, death, emigration, or the end of follow-up, whichever came first.

### Statistical analysis

To assess the relative risk of cancer among individuals with OCD, we used Cox proportional hazards models to calculate hazard ratios (HRs) and their 95% confidence intervals (CIs) of overall or site-specific cancers. For the full sibling analysis, we used the marginal between-within Cox model [31]. The proportional hazards assumption was evaluated using Schoenfeld residuals, with no major violations observed. Nonetheless, to further assess potential non-proportionality and evaluate whether the risk of cancer varied over time following the OCD diagnosis (e.g., due to early detection bias or reverse causality), we fitted flexible parametric survival models with time-varying covariate effects via restricted cubic splines for the Swedish cohort. To optimize computational efficiency when analyzing data of the large cohorts, Cox proportional hazards regression was used for primary relative risk analyses, while flexible parametric models [32] were also utilized to estimate absolute risk metrics. Specifically, we calculated standardized cumulative risk (1 − survival) and risk differences at age 65 using the G-formula [33], across the population-based matched cohorts. The age of 65 was selected because many of the cancers under study have a higher incidence at older ages. However, as risk sets naturally decline at older attained ages, estimates near age 65 are subject to wider CIs and reflect model-based parametric projections rather than crude empirical incidence. For the restricted cubic spline of the baseline hazard, we placed the knots at standard default 0th, 33rd, 67th, and 100th of the log (uncensored survival time). The analyses of the population-based matched cohorts were adjusted for sex, birth year, calendar time, and parental history of mental illness or cancer, while the analyses of the sibling-matched cohorts were adjusted for sex, birth year, calendar time, and family identity. Lastly, pooled estimates were derived using fixed-effect meta-analysis given the low between-country heterogeneity and relatively harmonized register infrastructure. As a sensitivity analysis, all models were re-estimated using random-effects meta-analysis to evaluate whether potential between-population variance influenced the pooled CIs. In the Sweden-only sensitivity analyses, we further adjusted for history of mental illness of the index persons other than OCD (diagnosed before the cohort entry date), family income, and highest parental education level. Missing values for educational level and family income were classified into a separate “unknown” category. Lastly, to account for all-cause mortality as a competing risk, we used flexible parametric survival models to jointly estimate cause-specific hazards for cancer diagnosis and pre-cancer death. From these models, we derived cumulative incidence functions (CIF) to estimate the true risk of cancer while adjusting for the competing risk of death.

For the analyses of cancer-specific and all-cause mortality, we used flexible parametric survival models (using the same knot placement). The models were adjusted for sex, birth year, age at cancer diagnosis, and type of cancer (i.e., specific type of primary cancer at diagnosis) to account for the vastly different baseline mortality and relapse profiles across distinct malignancies.

All statistical analyses were conducted in R software (R 4.4.3). A two-sided *p* < 0.05 was considered statistically significant.

## Results

### Descriptive statistics

The population-based matched cohorts included a total of 1,202,019 individuals, of which 57,239 had an OCD diagnosis. A total of 56.6% and 60.2% were females in Sweden and Denmark, respectively (Table 1). During a median follow-up time of 6.5 and 5.9 years, respectively, a total of 31,748 cancer cases were identified, including 21,712 in Sweden and 10,036 in Denmark. A higher percentage of individuals with OCD had a parental history of mental illness compared with their OCD-free counterparts in both countries, whereas the prevalence of parental history of cancer was relatively similar between groups. Similar characteristics were found for the sibling-matched cohorts (Additional file 1: Table S4). Numbers of cases of site-specific cancers are provided in ‘Additional file 1: Tables S5 and S6’.

**Table 1:** Baseline characteristics of the population-based matched cohorts in Sweden and Denmark

Characteristics	Sweden		Denmark	
	Individuals with OCD (*N* = 39,963)	Matched unexposed individuals (*N* = 799,260)	Individuals with OCD (*N* = 17,276)	Matched unexposed individuals (*N* = 345,520)
Female, N (%)	22,619 (56.6)	452,380 (56.6)	10,394 (60.2)	207,880 (60.2)
Birth year, median (range)	1982 (1932–2010)	1982 (1932–2010)	1986 (1932–2009)	1986 (1932–2009)
Parental history of mental illness, N (%)	11,961 (29.9)	156,590 (19.6)	5,071 (29.4)	72,364 (20.9)
Parental history of cancer, N (%)	6,145 (15.4)	124,253 (15.5)	2,295 (13.3)	46,225 (13.4)
Follow-up in years, median (IQR)	6.5 (7.6)	6.6 (7.7)	5.9 (8.5)	5.9 (8.6)
Age at cohort entry in years, median (IQR)	25.9 (18.5)	25.9 (18.5)	21.7 (17.2)	21.7 (17.2)
No. of incident cancer cases (%)	944 (2.4)	20,768 (2.6)	428 (2.5)	9,608 (2.8)
**Family income, N (%)**				
Bottom 20%	11,701 (29.3)	121,411 (15.2)	*NA*	*NA*
Middle	19,404 (48.6)	440,385 (55.1)	*NA*	*NA*
Top 20%	6,197 (15.5)	178,521 (22.3)	*NA*	*NA*
Unknown	2,661 (6.7)	58,943 (7.4)	*NA*	*NA*
**Parental education level, N (%)**				
Elementary	4,436 (11.1)	88,098 (11.0)	*NA*	*NA*
Secondary	14,535 (36.4)	301,036 (37.7)	*NA*	*NA*
Higher	13,546 (33.9)	261,271 (32.7)	*NA*	*NA*
Unknown	7,446 (18.6)	148,855 (18.6)	*NA*	*NA*

### OCD and risk of cancer morbidity

**Fig. 1:**
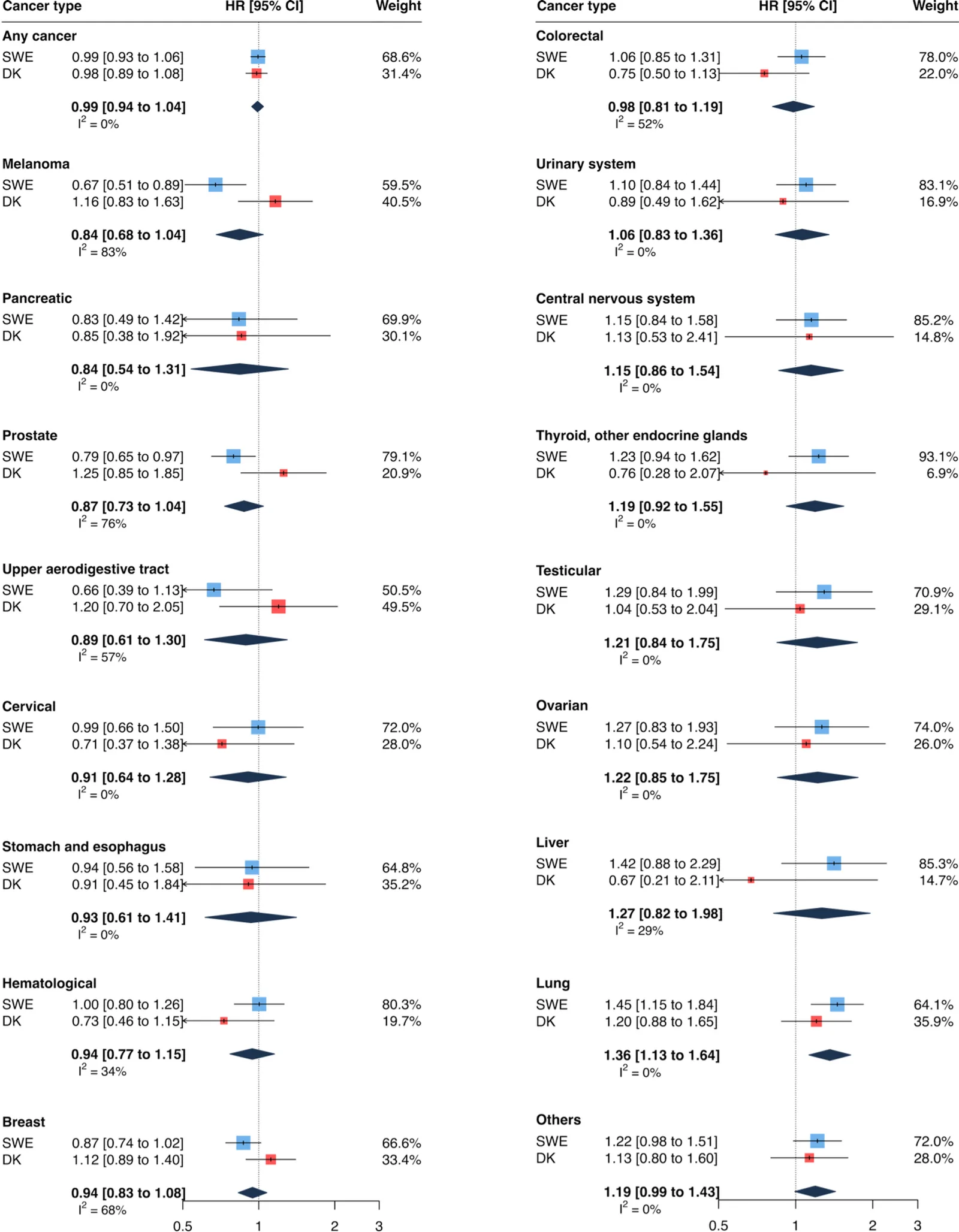
Hazard ratios (HRs) and 95% confidence intervals (95% CIs) for overall cancer and site-specific cancers in relation to obsessive-compulsive disorder. Estimates were derived from Cox proportional hazards models adjusted for sex, birth year, calendar time, and parental history of mental illness or cancer. Combined estimates were derived from fixed-effects meta-analysis. I^2^: Heterogeneity, SWE: Sweden, DK: Denmark

In the population-based matched cohorts, OCD was not associated with an increased risk of overall cancer (pooled HR: 0.99, 95% CI: 0.94–1.04, *p* = 0.69) (Fig. 1). However, for site-specific cancers, individuals with OCD had an increased risk of lung cancer, compared with their unexposed counterparts (pooled HR: 1.36, 95% CI: 1.13–1.64, *p* < 0.01), after adjusting for sex, birth year, calendar time, and parental history of mental illness and cancer (Fig. 1). In a further analysis in Sweden, additionally adjusting for history of other mental illness of the index persons (56.4% for individuals with OCD, compared with 11% for those without), family income, and parental education, the association with lung cancer was attenuated to the null (HR: 0.96, 95% CI: 0.75–1.24, *p* = 0.77) (Additional file 1: Table S7). In sensitivity analyses using random-effects meta-analysis, pooled HRs were virtually identical to the primary fixed-effect models (Additional file 1: Tables S8 and S9). As expected, CIs were slightly wider only for specific cancer types exhibiting modest between-country heterogeneity, with no changes to overall results. Moreover, accounting for the competing risk of death did not alter the overall findings, providing evidence that the observed results were not driven by differential mortality (Additional file 1: Fig. S1). Finally, in flexible parametric survival models evaluating time-varying HRs for overall cancer in the Swedish cohort, the estimated relative risk remained consistent with the null throughout follow-up. Although there appeared to be an increased risk during the first few years following OCD diagnosis, the risk increase was not statistically significant due to the small number initial events and the subsequently wide 95% CIs (Additionally file 1: Fig. S2).

Most site-specific cancers showed similar results between Sweden and Denmark (heterogeneity I^2^ test < 50%) (Fig. 1). The exceptions were melanoma and prostate cancer: in Sweden, OCD was associated with a lower risk of melanoma (HR: 0.67, 95% CI: 0.51–0.89, *p* < 0.01) and prostate cancer (HR: 0.79, 95% CI: 0.65–0.97, *p* = 0.02), whereas no association was observed in Denmark (melanoma: HR: 1.16, 95% CI: 0.83–1.63, *p* = 0.37; prostate cancer: HR: 1.25, 95% CI: 0.85–1.85, *p* = 0.25). In the sensitivity analysis in Sweden, the association for melanoma remained (HR: 0.72, 95% CI: 0.54–0.97, *p* = 0.03), while the association for prostate cancer attenuated to the null (HR: 0.88, 95% CI: 0.71–1.08, *p* = 0.21) after additional adjustments (Additional file 1: Table S7).

**Fig. 2:**
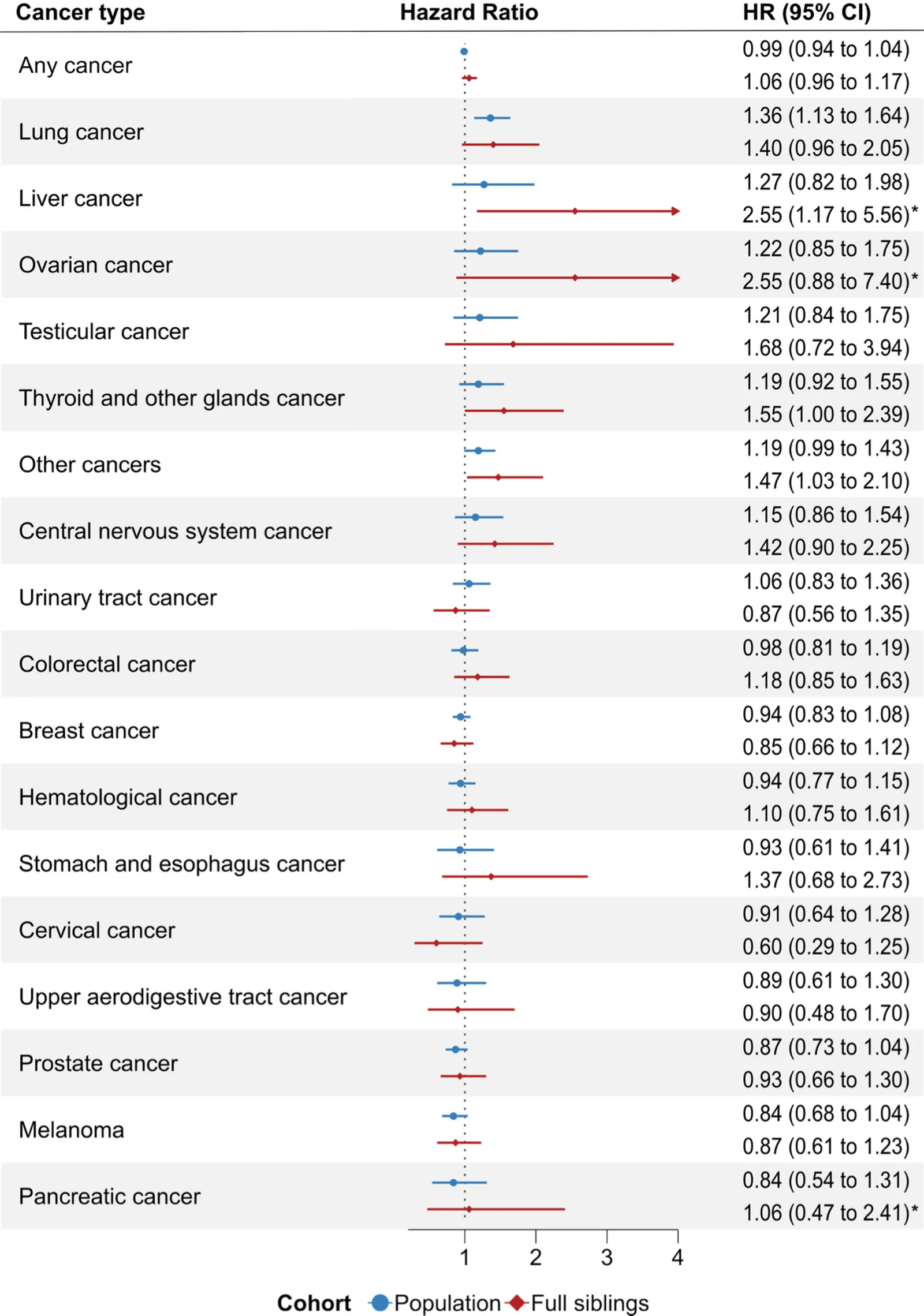
Hazard ratios (HRs) and 95% confidence intervals (95% CIs) for overall cancer and site-specific cancers in relation to obsessive-compulsive disorder - results from population-based matched cohorts and sibling-matched cohorts. Pooled estimates from Sweden and Denmark were derived from Cox proportional hazards models after adjustment for sex, birth year, calendar time, and parental history of mental illness or cancer in the population-based matched cohorts, and for sex and birth year in the sibling-matched cohorts. Meta-analyzed estimates were derived from fixed-effects meta-analysis. *Estimates were derived only from Sweden due to insufficient numbers in Denmark for model stability

The pooled results from the sibling-matched cohorts were largely consistent with those from the population-based matched cohorts (Fig. 2, Additional file 1: Table S10). Individuals with OCD did not have an increased risk of overall cancer, compared with their unexposed full siblings (pooled HR: 1.06, 95% CI: 0.96–1.17, *p* = 0.22). The positive association with lung cancer was consistent in sibling analysis, albeit it was not statistically significant (pooled HR: 1.40, 95% CI: 0.96–2.05, *p* = 0.08). Moreover, based on a limited number of cases in Sweden, individuals with OCD showed a higher risk of liver cancer, compared with their unexposed full siblings (HR: 2.55, 95% CI: 1.17–5.56, *p* = 0.02). The Danish cohort provided insufficient case counts for model stability.

### Standardized cumulative risk at age 65

**Fig. 3:**
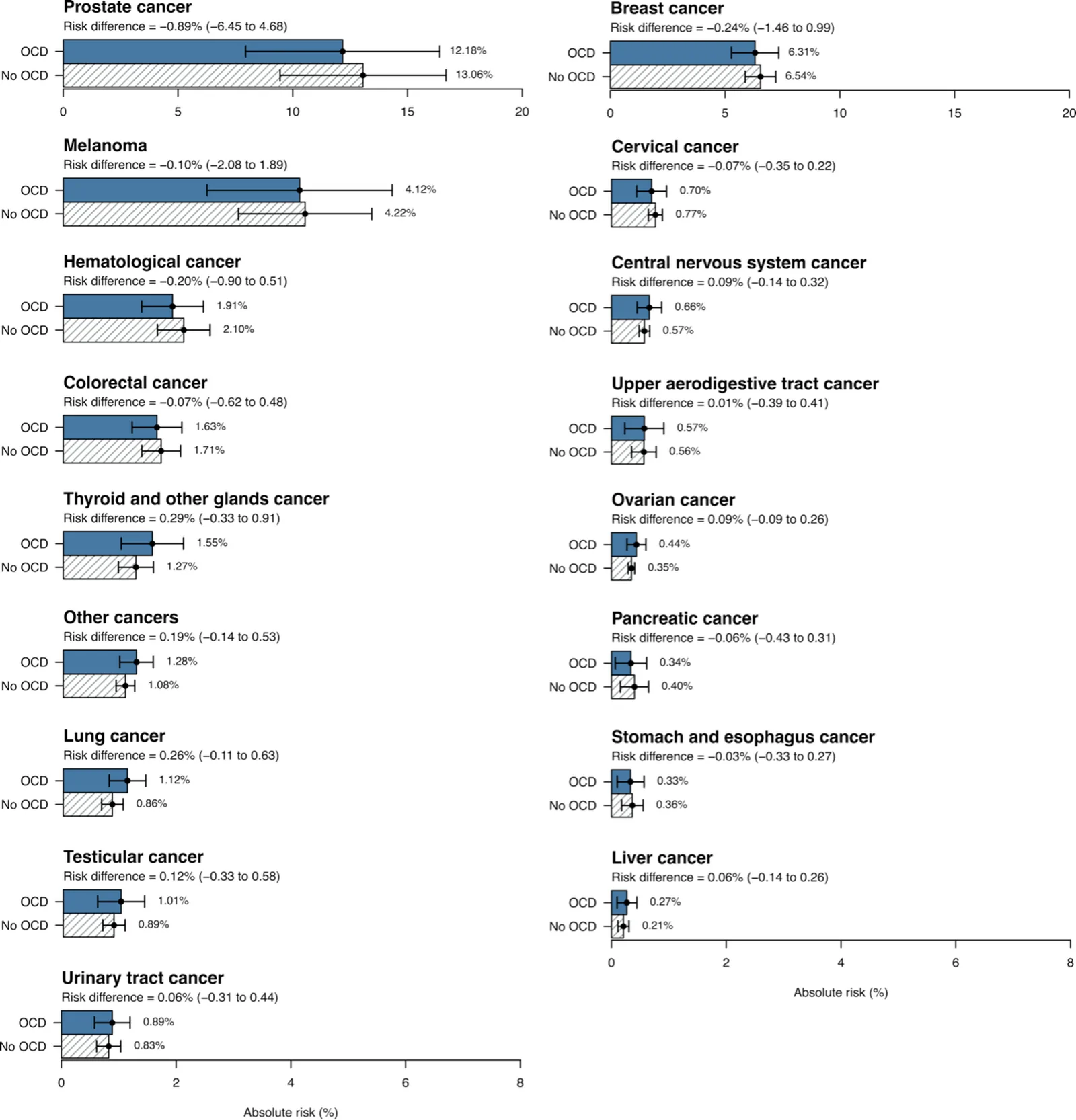
Standardized cumulative risk (1-survival) % and risk difference % (95% CI) between individuals with and without obsessive-compulsive disorder at age 65 for overall cancer and site-specific cancers in the population-based matched cohorts of Sweden and Denmark. Estimates were derived after standardization (G-formula) from flexible parametric survival models and adjusted for sex, birth year, calendar time, and parental history of mental illness or cancer. Meta-analyzed estimates were derived from fixed-effects meta-analysis. Weights were taken from the Cox models

To quantify cancer risk following an OCD diagnosis, we meta-analyzed standardized cumulative risks and risk differences at age 65 across both countries (Fig. 3; Additional file 1: Fig. S3 and S4). Overall, none of the site-specific cancers showed significant differences in the meta-analyzed absolute risks, which remained relatively low for most site-specific cancers in both groups due to the low incidence of most cancers. When looking into country-specific estimates, a reduced cumulative risk of 0.86% (95% CI: 0.02–1.70) was noted for melanoma in individuals with OCD in Sweden (Additional file 1: Fig. S3).

### Mortality risk after cancer diagnosis in Sweden

**Fig. 4:**
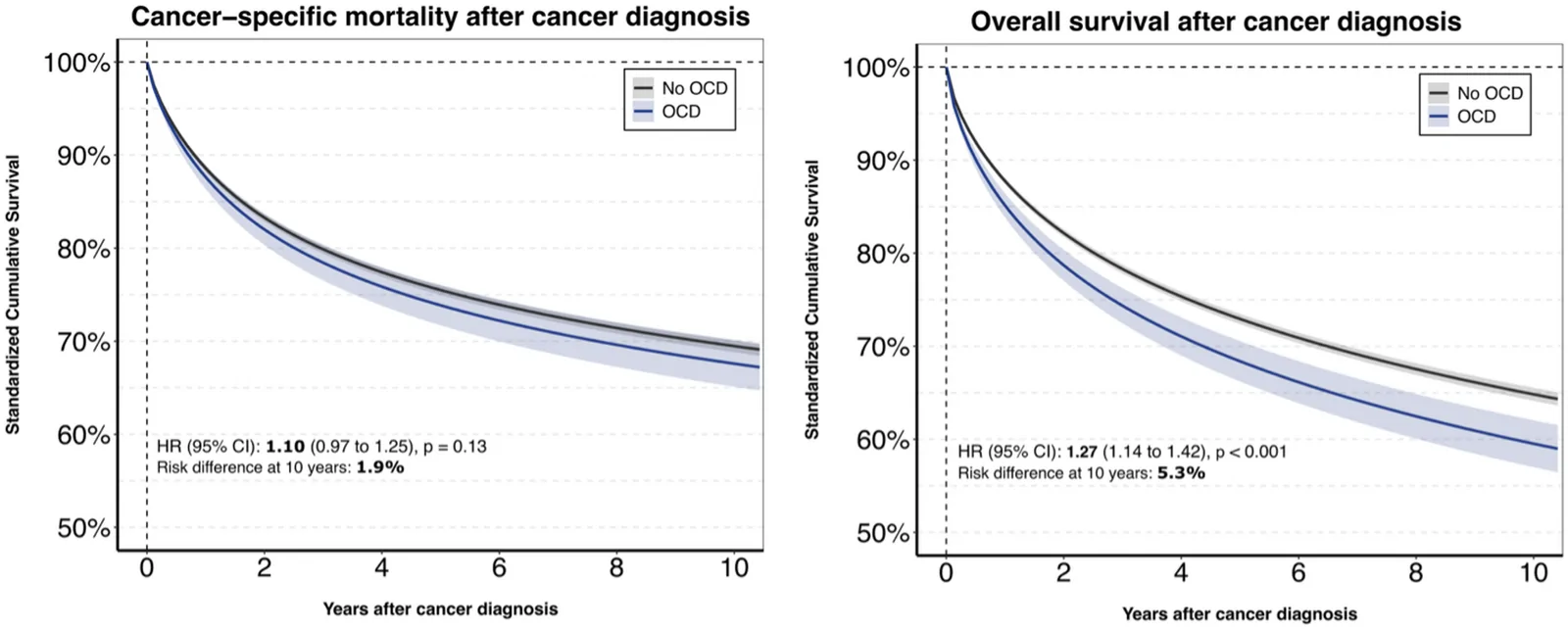
Cancer-specific and overall survival after cancer diagnosis among individuals with and without obsessive-compulsive disorder in the population-based matched cohort of Sweden. Estimates were derived after standardization (G-formula) from flexible parametric survival models and adjusted for sex, birth year, age at cancer diagnosis, and type of cancer

Within the 21,712 individuals with a cancer diagnosis in the Swedish population-based matched cohort, there was no significant difference in cancer-specific mortality between those with and without OCD (HR: 1.10, 95% CI: 0.97–1.25, *p* = 0.13, 10-year survival difference: 1.9%) (Fig. 4). By contrast, individuals with OCD had a higher risk of all-cause mortality after a cancer diagnosis (HR: 1.27, 95% CI: 1.14–1.42, *p* < 0.001, 10-year survival difference: 5.3%). We could not conduct these analyses in Denmark due to limited numbers of outcomes in the cohort.

## Discussion

In this cohort study in Sweden and Denmark, through population-based and sibling matched designs, OCD was not robustly associated with an increased risk of cancer morbidity. Sensitivity analyses accounting for the competing risk of death in the Swedish cohort showed consistent results. While OCD was associated with increased risk of some specific cancers (i.e., lung cancer) and decreased risk of others (i.e., melanoma and prostate cancer), these results tended to attenuate after adjustment of additional covariates or in the sibling analysis accounting for familial confounding. Furthermore, in the Swedish cohort, OCD was associated with all-cause mortality, but not with cancer-specific mortality, following a cancer diagnosis.

Our findings are consistent with a Taiwanese nationwide register-based study which reported no association between OCD and overall cancer risk [16]. Extending this prior work, we conducted nationwide analyses in two countries and additionally implemented sibling comparisons to account for familial confounding. Despite differences in study populations, healthcare systems, diagnostic practices, analytic approaches, and covariates considered, the results remained largely consistent across these studies.

We found that the association between OCD and increased lung cancer risk was largely explained by socioeconomic factors and psychiatric comorbidities. Previous studies have shown that OCD is frequently co-occurrent with socioeconomic adversity [7] and the presence of other mental disorders (i.e., anxiety disorders, depression, substance use disorders) [4, 5, 9]. Individuals in lower socioeconomic groups and those with psychiatric disorders often have a higher smoking prevalence and increased exposure to environmental pollutants [34–36]. Therefore, the increased lung cancer risk in individuals with OCD might be better understood as a consequence of these underlying social and behavioral determinants rather than a direct impact of the OCD itself. Given that socioeconomic disadvantage is an established risk factor across multiple cancers [37], it could theoretically support a hypothesis of increased incidence for other cancer types as well. However, we did not observe a generalized increase in cancer incidence in our results. Although speculative, this discrepancy may be explained by several factors. First, specific behavioral manifestations of OCD, such as avoidance of specific toxins or environmental hazards, might counteract increment in cancer risks. Second, screening barriers or delayed healthcare engagement in people with psychiatric conditions may obscure true incidence by leaving early-stage cancers undiagnosed [3, 38]. As such, the complex interplay between potentially protective and harmful health behaviors in OCD may neutralize overall risk at the population level [3, 38].

Our findings in Sweden also suggested a decreased risk of melanoma and prostate cancer among individuals with OCD. Melanoma incidence has been increasing among individuals aged 60 and above in Sweden, while decreasing among those under 49 years of age [39]. One of the possible explanations for the observed lower risk of melanoma and prostate cancer among individuals with OCD might include lower detection, often seen in other psychiatric disorders such as depression [40, 41]. The literature on healthcare utilization in OCD is complex, showing both increased care-seeking and costs due to somatic hypervigilance and comorbidities and reduced utilization due to avoidance [42, 43]. While greater healthcare contact might theoretically increase cancer detection in OCD, our analyses showed null associations across both nationally screened cancers (e.g., cervical, breast, colorectal) and symptom-detected cancers. Because surveillance bias would typically produce positive associations in heavily screened cancers, these null findings indicate that healthcare utilization differences do not materially bias our estimates. Therefore, the lower rates of melanoma and prostate cancer are more consistent with specific detection barriers or underdiagnosis, leading to early-stage lesions remaining undetected until advanced stages. Future studies should investigate screening participation and cancer-related healthcare utilization patterns among individuals with OCD to further investigate the mechanism.

For some site-specific cancers, such as melanoma and prostate cancer, the findings in Sweden were not replicated in Denmark, despite similarities in both countries’ health care systems and broader cultural contexts [44, 45]. Nonetheless, the two countries have some differences in cancer diagnosis procedures (e.g., prostate cancer diagnoses rose in Sweden in the early 1990s due to prostate specific antigen screening, while in Denmark they rose in early 2000s with the inclusion of pathology register information) [46]. Additionally, there might be variations in how OCD is diagnosed and treated between the two countries. For instance, it has been shown that the prevalence of most psychiatric disorders and treatment density are lower in the Danish than in the Swedish specialist care [47]. This might indicate that milder cases of OCD might be predominantly treated in primary care in Denmark compared to Sweden. As we lacked information on primary care diagnoses, this can lead to varying severity of OCD cases identified between the two countries, resulting in distinct characteristics and, consequently, different cancer risks.

In our earlier study, individuals with OCD had an 82% higher risk of all-cause mortality and a lower risk of cancer-specific mortality, compared with individuals without OCD [17]. However, the association on cancer-specific mortality was attenuated in sibling analyses, suggesting that familial confounding may have contributed to the observed finding [17]. In the present study, we followed all individuals with an incident cancer diagnosis from the time of diagnosis to assess mortality outcomes. We found that individuals with OCD had a higher risk of all-cause mortality following a cancer diagnosis, whereas cancer-specific mortality did not differ materially between the groups. These findings suggest that, although risk of death directly related to cancer appears similar, individuals with OCD remain at increased risk of death from other causes after a cancer diagnosis. This persistent excess mortality may be driven by several mechanisms, including barriers to healthcare access and the burden of pre-existing somatic or psychiatric comorbidities, which may be overlooked during cancer care. Although cancer-specific mortality was not significantly elevated, suboptimal adherence to cancer therapies, which can contribute to all-cause mortality through, for example, treatment complications and co-occurring infections, may still play a contributory role. However, as cancer-specific mortality remained comparable, the overall excess mortality likely stems primarily from broader challenges in navigating healthcare systems, managing underlying non-cancer conditions, and the compounding impact of unmanaged somatic and psychiatric comorbidities. This excess mortality is consistent with prior evidence showing increased mortality due to both natural (e.g., somatic diseases) and unnatural (e.g., suicide, accidents) causes among individuals with OCD [17, 48], indicating the importance of comprehensive clinical management beyond cancer care alone.

### Strengths and limitations

The main strengths of our study include a relatively large sample size from two countries with high-quality national registers and the inclusion of an older population which provided sufficient statistical power to analyze rare cancer types. Additionally, OCD diagnoses have been validated in both countries’ national registers, showing high positive predictive value [28, 29]. Another strength is the use of a sibling analysis, which helped control for familial confounding due to genetic and early-life factors, as well as the calculation of absolute risks to have a more complete perspective of the risks. Finally, the comprehensive list of site-specific cancers included, allowed us to assess in detail how the risk of developing specific cancers differs in individuals with OCD.

Several limitations to this study should be noted. We lacked information on lifestyle factors such as smoking, physical activity, and diet, which may influence the association between OCD and cancer risk. Additionally, we lacked data on educational attainment and income in Denmark, so these variables were not included in the main analysis. However, such data were available in Sweden and were adjusted for in sensitivity analyses. Moreover, we did have limited power for some analyses (sibling and the cumulative risk analyses) due to few cases for rare cancer types (i.e., pancreatic and liver cancers), especially in Denmark. Considering the OCD ascertainment, we did not have access to primary care data, and as a result, our sample may primarily reflect more severe cases of OCD in both countries. Another consideration is the shift from ICD-8, ICD-9 to ICD-10 across the study period. ICD-8 was primarily used in both countries before the inclusion of outpatient data (1995 in Denmark and 2001 in Sweden); nonetheless, the vast majority of OCD cases in the present study were ascertained through ICD-10 codes and outpatient care. There is also a risk that some individuals in the comparison groups might have undiagnosed OCD, which could potentially bias our estimates toward the null. Similarly, although both the Swedish and Danish cancer registers have been shown to have overall excellent sensitivity in cancer ascertainment, the coverage varies across cancer types (e.g., coverage of liver cancer can be around 60–65% in Sweden) [49]. Additionally, our results may primarily be generalizable to Nordic countries or countries with similar healthcare systems and socioeconomic statuses. Lastly, because individuals with OCD have higher all-cause mortality [17], survival bias could lower cancer detection rates. While our competing risk analysis showed that death did not drive the findings for overall cancer, residual survival bias may still be present, especially across individual cancer subtypes.

## Conclusions

Using complementary population-based and sibling-matched cohorts based on nationwide register data from Sweden and Denmark, we found little evidence that OCD was associated with overall cancer risk or cancer-specific mortality. However, OCD was associated with a higher risk of all-cause mortality after cancer diagnosis.

## Supplementary Information

Below is the link to the electronic supplementary material.

Supplementary Material 1

## Data Availability

The individual-level register data used in this study are not publicly available due to data protection laws in each participating country and GDPR. Data from Sweden can be obtained through a formal application to the National Board of Health and Welfare (Socialstyrelsen) for health registers and Statistics Sweden for socioeconomic and demographic variables. Data from Denmark are available via application to the Danish Health Data Authority (Sundhedsdatastyrelsen).

## Abbreviations

OCD: Obsessive-compulsive disorder
LISA: The longitudinal integrated database for health insurance and labour market studies
ICD: International classification of diseases
