# Supplementary material for: Cancer morbidity and mortality in individuals with obsessive-compulsive disorder: a population-based and sibling cohort study in Sweden and Denmark
Source: BMC Med. 2026 Nov 10;24:634. doi: 10.1186/s12916-026-05386-7 (PMC13756859; doi:10.1186/s12916-026-05386-7)
Supplement: Supplementary file 1 [file 12916_2026_5386_MOESM1_ESM.docx]

**Additional file 1**

| Table S1. Description of registries in Sweden and Denmark and underlying differences in coverage years | | | |
| --- | --- | --- | --- |
| Register | **Sweden** |  | **Denmark** |
| National Patient Register | Inpatient care since 1973 (for psychiatric disorders), and all specialized outpatient care since 2001. |  | Inpatient care since 1978, and all specialized outpatient care since 1995 |
| The Danish Psychiatric Central Research Register | - |  | All psychiatric hospital admissions from 1969 onwards and outpatient care since 1995 |
| Cancer Register | Almost complete records since 1958 |  | Almost complete records since 1943 |

| **Table S2.** STROBE checklist of items that should be included in reports of cohort studies. | | | |
| --- | --- | --- | --- |
|  | Item No | Recommendation | Page No |
| **Title and abstract** | 1 | (*a*) Indicate the study’s design with a commonly used term in the title or the abstract | 1 |
|  |  | (*b*) Provide in the abstract an informative and balanced summary of what was done and what was found | 3 |
| Introduction | | | |
| Background/rationale | 2 | Explain the scientific background and rationale for the investigation being reported | 4-5 |
| Objectives | 3 | State specific objectives, including any prespecified hypotheses | 5 |
| Methods | | | |
| Study design | 4 | Present key elements of study design early in the paper | 8-9 |
| Setting | 5 | Describe the setting, locations, and relevant dates, including periods of recruitment, exposure, follow-up, and data collection | 8-9 |
| Participants | 6 | (*a*) Give the eligibility criteria, and the sources and methods of selection of participants. Describe methods of follow-up | 6, 8-9 |
|  |  | (*b*) For matched studies, give matching criteria and number of exposed and unexposed | 8-9 |
| Variables | 7 | Clearly define all outcomes, exposures, predictors, potential confounders, and effect modifiers. Give diagnostic criteria, if applicable | 6-8 |
| Data sources/ measurement | 8* | For each variable of interest, give sources of data and details of methods of assessment (measurement). Describe comparability of assessment methods if there is more than one group | 6-8 |
| Bias | 9 | Describe any efforts to address potential sources of bias | 9-10 |
| Study size | 10 | Explain how the study size was arrived at | 8-9, 10-11 |
| Quantitative variables | 11 | Explain how quantitative variables were handled in the analyses. If applicable, describe which groupings were chosen and why | 7-8, 12 |
| Statistical methods | 12 | (*a*) Describe all statistical methods, including those used to control for confounding | 9-10 |
|  |  | (*b*) Describe any methods used to examine subgroups and interactions | 9-10 |
|  |  | (*c*) Explain how missing data were addressed | 10 |
|  |  | (*d*) If applicable, explain how loss to follow-up was addressed | 8-9 |
|  |  | (*e*) Describe any sensitivity analyses | 9-10 |
| Results | | |  |
| Participants | 13* | (a) Report numbers of individuals at each stage of study—eg numbers potentially eligible, examined for eligibility, confirmed eligible, included in the study, completing follow-up, and analysed | 10-12 |
|  |  | (b) Give reasons for non-participation at each stage | NA |
|  |  | (c) Consider use of a flow diagram | NA |
| Descriptive data | 14* | (a) Give characteristics of study participants (eg demographic, clinical, social) and information on exposures and potential confounders | 11-12, 6 (Suppl.) |
|  |  | (b) Indicate number of participants with missing data for each variable of interest | 11-12 |
|  |  | (c) Summarise follow-up time (eg, average and total amount) | 11-12, 6 (Suppl.) |
| Outcome data | 15* | Report numbers of outcome events or summary measures over time | 11-12, 7-8 (Suppl.) |

| Main results | 16 | (*a*) Give unadjusted estimates and, if applicable, confounder-adjusted estimates and their precision (eg, 95% confidence interval). Make clear which confounders were adjusted for and why they were included | 12-19 |
| --- | --- | --- | --- |
|  |  | (*b*) Report category boundaries when continuous variables were categorized | 12 |
|  |  | (*c*) If relevant, consider translating estimates of relative risk into absolute risk for a meaningful time period | 17-18 |
| Other analyses | 17 | Report other analyses done—eg analyses of subgroups and interactions, and sensitivity analyses | 9-11 (Suppl.), 13-14 (Suppl.) |
| Discussion | | | |
| Key results | 18 | Summarise key results with reference to study objectives | 19-20 |
| Limitations | 19 | Discuss limitations of the study, taking into account sources of potential bias or imprecision. Discuss both direction and magnitude of any potential bias | 23-24 |
| Interpretation | 20 | Give a cautious overall interpretation of results considering objectives, limitations, multiplicity of analyses, results from similar studies, and other relevant evidence | 19-23 |
| Generalisability | 21 | Discuss the generalisability (external validity) of the study results | 24 |
| Other information | | | |
| Funding | 22 | Give the source of funding and the role of the funders for the present study and, if applicable, for the original study on which the present article is based | 25 |

*Give information separately for exposed and unexposed groups.

**Note:** An Explanation and Elaboration article discusses each checklist item and gives methodological background and published examples of transparent reporting. The STROBE checklist is best used in conjunction with this article (freely available on the Web sites of PLoS Medicine at http://www.plosmedicine.org/, Annals of Internal Medicine at http://www.annals.org/, and Epidemiology at http://www.epidem.com/). Information on the STROBE Initiative is available at http://www.strobe-statement.org.

| Table S3. ICD codes used to identify cancer types in Sweden and Denmark | | |
| --- | --- | --- |
| Cancer type | **ICD-7 version*** | **ICD-10 version^†^** |
| Any cancer | 140-207 | C00-C97, D45, D46, D471, D473 |
| Breast | 170 | C50 |
| Central nervous system | 193 | C70-C72 |
| Cervical | 171 | C53 |
| Colorectal | 153-154 | C18-C20 |
| Hematological | 200-207 | C81-C96, D45, D46, D471, D473 |
| Liver and biliary passages | 155 | C22-C24 |
| Lung, trachea, bronchus | 162-163 | C33-C34 |
| Melanoma | 190 | C43 |
| Ovarian | 1750 | C56 |
| Pancreatic | 157 | C25 |
| Prostate | 177 | C61 |
| Stomach and esophagus | 150-151 | C15, C16 |
| Thyroid and other endocrine glands | 194-195 | C73-C75 |
| Testicular | 178 | C62 |
| Upper aerodigestive tract (lip, oral cavity, nose, nasal and accessory sinuses pharynx and larynx) | 140-148, 160-161 | C00-C14, C30-C32 |
| Urinary tract | 180-181 | C64-C68 |
| Other types | 152, 156, 158, 159, 164,165,179, 192, 196-199 | C17, C21, C26, C37-C39, C40-C41, C45-C49, C60, C63, C69, C76-C80, C97 |

Skin (excl. melanoma) is not included as a separate category due to diagnostic irregularities between the two countries.
*****ICD-7 codes are still used in the Swedish Cancer register. In Denmark they were used from 1943-1977.
**^†^**ICD-10 codes are officially used in Denmark since 2003. From 1978-2003, ICD-7 codes were converted to ICD-10.

| Table S4. Baseline characteristics of the sibling-matched cohorts in Sweden and Denmark | | | | | |
| --- | --- | --- | --- | --- | --- |
|  | **Sweden** | |  | **Denmark** | |
| Characteristic | Individuals with OCD (N = 25,501) | OCD-free matched full siblings  (N = 39,445) |  | Individuals with OCD (N = 11,483) | OCD-free matched full siblings  (N = 15,670) |
| Female, N (%) | 14,613 (57.3) | 19,361 (49.1) |  | 6,895 (60.0) | 7,707 (49.2) |
| Birth year,  Median (range) | 1983 (1932-2010) | 1980 (1932-2010) |  | 1988 (1935-2009) | 1987 (1932-2009) |
| Follow-up in years,  Median (IQR) | 6.7 (7.7) | 7.0 (7.8) |  | 5.5 (7.8) | 5.6 (7.9) |
| Age at OCD diagnosis in years,  Median (IQR) | 25.7 (18.2) | - |  | 20.5 (14.4) | - |

| Table S5. Number of cancer cases for the population-based matched cohorts in Sweden and Denmark | | | | | |
| --- | --- | --- | --- | --- | --- |
|  | **Sweden** | |  | **Denmark** | |
| Cancer type, N (%) | Individuals with OCD (N = 39,963) | OCD-free matched individuals (N = 799,260) |  | Individuals with OCD (N = 17,276) | OCD-free matched individuals (N = 345,520) |
| All types | 944 (2.4) | 20,768 (2.6) |  | 428 (2.5) | 9,608 (2.8) |
| Breast | 154 (0.4) | 3,746 (0.5) |  | 78 (0.5) | 1,519 (0.4) |
| Central nervous system | 41 (0.1) | 761 (0.1) |  | 7 (0.0) | 137 (0.0) |
| Cervical | 24 (0.1) | 484 (0.1) |  | 9 (0.1) | 262 (0.1) |
| Colon and rectum | 84 (0.2) | 1,788 (0.2) |  | 25 (0.1) | 735 (0.2) |
| Hematological | 78 (0.2) | 1,697 (0.2) |  | 19 (0.1) | 583 (0.2) |
| Liver and biliary passages | 18 (0.0) | 284 (0.0) |  | ≤ 5 (0.0) | 103 (0.0) |
| Lung, trachea, bronchus | 75 (0.2) | 1,153 (0.1) |  | 41 (0.2) | 778 (0.2) |
| Melanoma | 51 (0.1) | 1,597 (0.2) |  | 36 (0.2) | 657 (0.2) |
| Ovarian | 23 (0.1) | 382 (0.0) |  | 8 (0.0) | 156 (0.0) |
| Pancreatic | 14 (0.0) | 372 (0.0) |  | 6 (0.0) | 167 (0.0) |
| Prostate | 104 (0.3) | 3,028 (0.4) |  | 27 (0.2) | 535 (0.2) |
| Stomach and esophagus | 15 (0.0) | 364 (0.0) |  | 8 (0.0) | 209 (0.1) |
| Testicular | 22 (0.1) | 339 (0.0) |  | 9 (0.0) | 177 (0.1) |
| Theroid and other glands | 55 (0.1) | 926 (0.1) |  | ≤ 5 (0.0) | 108 (0.0) |
| Upper aerodigestive tract | 14 (0.0) | 459 (0.1) |  | 15 (0.1) | 260 (0.1) |
| Urine tract | 56 (0.1) | 1,158 (0.1) |  | 11 (0.1) | 287 (0.1) |
| Other | 86 (0.2) | 1,544 (0.2) |  | 34 (0.2) | 679 (0.2) |

| Table S6. Number of cancer cases for the sibling-matched cohorts in Sweden and Denmark | | | | | |
| --- | --- | --- | --- | --- | --- |
|  | **Sweden** | |  | **Denmark** | |
| Cancer type, N (%) | Individuals with OCD (N = 25,501) | OCD-free matched siblings (N = 39,445) |  | Individuals with OCD (N = 11,483) | OCD-free matched siblings (N = 15,670) |
| All types | 581 (2.3) | 1,123 (2.8) |  | 137 (1.2) | 232 (1.5) |
| Breast | 96 (0.4) | 192 (0.5) |  | 20 (0.2) | 38 (0.2) |
| Central nervous system | 30 (0.1) | 42 (0.1) |  | ≤ 5 (0.0) | ≤ 5 (0.0) |
| Cervical | 14 (0.1) | 26 (0.1) |  | 7 (0.1) | 8 (0.1) |
| Colon and rectum | 53 (0.2) | 108 (0.3) |  | 8 (0.1) | 11 (0.1) |
| Hematological | 46 (0.2) | 81 (0.2) |  | ≤ 5 (0.0) | 12 (0.1) |
| Liver and biliary passages | 14 (0.1) | 13 (0.0) |  | - | - |
| Lung, trachea, bronchus | 41 (0.2) | 69 (0.2) |  | ≤ 5 (0.0) | 14 (0.1) |
| Melanoma | 34 (0.1) | 82 (0.2) |  | 18 (0.2) | 16 (0.1) |
| Ovarian | 16 (0.1) | 12 (0.0) |  | ≤ 5 (0.0) | ≤ 5 (0.0) |
| Pancreatic | 10 (0.0) | 22 (0.1) |  | - | ≤ 5 (0.0) |
| Prostate | 61 (0.2) | 194 (0.5) |  | 6 (0.1) | 10 (0.1) |
| Stomach and esophagus | 11 (0.0) | 18 (0.0) |  | ≤ 5 (0.0) | ≤ 5 (0.0) |
| Testicular | 15 (0.1) | 14 (0.0) |  | ≤ 5 (0.0) | 10 (0.1) |
| Theroid and other glands | 37 (0.1) | 39 (0.1) |  | ≤ 5 (0.0) | ≤ 5 (0.0) |
| Upper aerodigestive tract | 9 (0.0) | 23 (0.1) |  | 6 (0.1) | 8 (0.1) |
| Urine tract | 27 (0.1) | 76 (0.2) |  | ≤ 5 (0.0) | 9 (0.1) |
| Other | 47 (0.2) | 67 (0.2) |  | 10 (0.1) | 10 (0.1) |

| Table S7. HRs with 95% CI for overall cancer and site-specific cancers among individuals with and without OCD in Sweden, additionally adjusted for highest parental education, household income, and history of any mental illness of the index persons | | | |  |
| --- | --- | --- | --- | --- |
| Cancer types, N (%) | Individuals with OCD (N = 39,963) | OCD-free matched individuals (N = 799,260) | HR (95% CI)^†^ | |
| All types | 944 (2.4) | 20,768 (2.6) | 0.95 (0.89-1.02) | |
| Breast | 154 (0.4) | 3,746 (0.5) | 0.94 (0.79-1.11) | |
| Central nervous system | 41 (0.1) | 761 (0.1) | 1.20 (0.85-1.67) | |
| Cervical | 24 (0.1) | 484 (0.1) | 0.73 (0.47-1-12) | |
| Colon and rectum | 84 (0.2) | 1,788 (0.2) | 1.06 (0.84-1.34) | |
| Hematological | 78 (0.2) | 1,697 (0.2) | 1.05 (0.82-1.33) | |
| Liver and biliary passages | 18 (0.0) | 284 (0.0) | 0.86 (0.51-1.44) | |
| Lung, trachea, bronchus | 75 (0.2) | 1,153 (0.1) | 0.96 (0.75-1.24) | |
| Melanoma | 51 (0.1) | 1,597 (0.2) | **0.72 (0.54-0.97)** | |
| Ovarian | 23 (0.1) | 382 (0.0) | 1.26 (0.80-1.99) | |
| Pancreatic | 14 (0.0) | 372 (0.0) | 0.71 (0.40-1.25) | |
| Prostate | 104 (0.3) | 3,028 (0.4) | 0.88 (0.71-1.08) | |
| Stomach and esophagus | 15 (0.0) | 364 (0.0) | 0.81 (0.47-1.40) | |
| Testicular | 22 (0.1) | 339 (0.0) | 1.10 (0.69-1.73) | |
| Theroid and other glands | 55 (0.1) | 926 (0.1) | 1.18 (0.88-1.58) | |
| Upper aerodigestive tract | 14 (0.0) | 459 (0.1) | **0.41 (0.24-0.72)** | |
| Urine tract | 56 (0.1) | 1,158 (0.1) | 1.03 (0.77-1.37) | |
| Other | 86 (0.2) | 1,544 (0.2) | 1.13 (0.89-1.43) | |

Derived from Cox proportional hazards models.
^†^Adjusted for sex, birth year, calendar time, parental history of mental disorders and cancer at the start of follow-up, household income, highest parental educational attainment, and history of any mental illness of the index persons

| Table S8. HRs for overall and site-specific cancers among individuals with OCD in the population-based matched cohorts in Sweden and Denmark, using random-effects meta-analysis. | | | | | |
| --- | --- | --- | --- | --- | --- |
| Cancer types | Sweden  HR (95% CI) | Denmark  HR (95% CI) | Pooled  HR (95% CI) | Heterogeneity (I^2^) | P-value |
| All types | 0.99 (0.93-1.06) | 0.98 (0.89-1.08) | 0.99 (0.94-1.04) | 0.00 | 0.69 |
| Breast | 0.87 (0.74-1.02) | 1.12 (0.89-1.40) | 0.97 (0.76-1.24) | 0.68 | 0.82 |
| Central nervous system | 1.16 (0.84-1.58) | 1.13 (0.53-2.41) | 1.15 (0.86-1.54) | 0.00 | 0.34 |
| Cervical | 1.00 (0.66-1.50) | 0.71 (0.37-1.38) | 0.91 (0.64-1.29) | 0.00 | 0.59 |
| Colon and rectum | 1.05 (0.85-1.31) | 0.75 (0.50-1.13) | 0.93 (0.68-1.28) | 0.50 | 0.66 |
| Hematological | 1.00 (0.80-1.26) | 0.73 (0.46-1.15) | 0.91 (0.69-1.21) | 0.33 | 0.53 |
| Liver and biliary passages | 1.42 (0.88-2.29) | 0.67 (0.21-2.11) | 1.18 (0.62-2.22) | 0.29 | 0.62 |
| Lung, trachea, bronchus | 1.46 (1.15-1.84) | 1.21 (0.88-1.65) | 1.36 (1.13-1.64) | 0.00 | <0.01 |
| Melanoma | 0.67 (0.51-0.89) | 1.17 (0.83-1.63) | 0.88 (0.51-1.51) | 0.84 | 0.64 |
| Ovarian | 1.27 (0.83-1.93) | 1.10 (0.54-2.24) | 1.22 (0.85-1.76) | 0.00 | 0.28 |
| Pancreatic | 0.83 (0.49-1.42) | 0.85 (0.38-1.92) | 0.84 (0.54-1.31) | 0.00 | 0.43 |
| Prostate | 0.79 (0.65-0.97) | 1.26 (0.85-1.85) | 0.97 (0.62-1.51) | 0.77 | 0.89 |
| Stomach and esophagus | 0.94 (0.56-1.58) | 0.91 (0.45-1.84) | 0.93 (0.61-1.41) | 0.00 | 0.73 |
| Testicular | 1.29 (0.84-1.99) | 1.04 (0.53-2.04) | 1.21 (0.84-1.74) | 0.00 | 0.30 |
| Thyroid and other glands | 1.24 (0.94-1.62) | 0.76 (0.28-2.07) | 1.20 (0.92-1.56) | 0.00 | 0.18 |
| Upper aerodigestive tract | 0.66 (0.39-1.13) | 1.19 (0.70-2.05) | 0.89 (0.50-1.58) | 0.57 | 0.69 |
| Urine tract | 1.10 (0.84-1.44) | 0.89 (0.49-1.62) | 1.06 (0.83-1.36) | 0.00 | 0.64 |
| Other | 1.22 (0.98-1.51) | 1.13 (0.80-1.60) | 1.19 (0.99-1.43) | 0.00 | 0.06 |

Estimates were derived from Cox proportional hazards models adjusted for sex, birth year, calendar time, and parental history of mental illness or cancer.

| Table S9. HRs for overall and site-specific cancers among individuals with OCD in the sibling matched cohorts in Sweden and Denmark, using random-effects meta-analysis. | | | | | |
| --- | --- | --- | --- | --- | --- |
| Cancer types | Sweden  HR (95% CI) | Denmark  HR (95% CI) | Pooled  HR (95% CI) | Heterogeneity (I^2^) | P-value |
| All types | 1.08 (0.97-1.20) | 0.98 (0.79-1.22) | 1.06 (0.97-1.16) | 0.00 | 0.22 |
| Breast | 0.88 (0.66-1.17) | 0.58 (0.21-1.54) | 0.85 (0.64-1.12) | 0.00 | 0.25 |
| Central nervous system | 1.39 (0.86-2.26) | 1.71 (0.40-7.39) | 1.42 (0.90-2.25) | 0.00 | 0.14 |
| Cervical | 0.60 (0.26-1.42) | 0.58 (0.13-2.53) | 0.60 (0.29-1.25) | 0.00 | 0.17 |
| Colon and rectum | 1.10 (0.78-1.56) | 1.88 (0.74-4.78) | 1.19 (0.81-1.76) | 0.10 | 0.37 |
| Hematological | 1.21 (0.81-1.81) | 0.52 (0.17-1.60) | 0.97 (0.41-2.27) | 0.57 | 0.94 |
| Liver and biliary passages | 2.55 (1.17-5.56) | - | - | - | - |
| Lung, trachea, bronchus | 1.48 (0.99-2.22) | 0.89 (0.28-2.78) | 1.40 (0.96-2.05) | 0.00 | 0.08 |
| Melanoma | 0.75 (0.51-1.12) | 1.41 (0.67-2.98) | 0.95 (0.52-1.71) | 0.52 | 0.86 |
| Ovarian | 2.55 (0.88-7.40) | - | - | - | - |
| Pancreatic | 1.06 (0.47-2.41) | - | - | - | - |
| Prostate | 0.90 (0.63-1.27) | 1.71 (0.40-7.33) | 0.93 (0.66-1.31) | 0.00 | 0.67 |
| Stomach and esophagus | 1.58 (0.71-3.49) | 0.89 (0.22-3.57) | 1.37 (0.69-2.73) | 0.00 | 0.37 |
| Testicular | 2.84 (0.99-8.11) | 0.63 (0.15-2.68) | 1.46 (0.34-6.28) | 0.63 | 0.61 |
| Thyroid and other glands | 1.70 (1.08-2.68) | 0.54 (0.12-2.43) | 1.21 (0.43-3.39) | 0.51 | 0.72 |
| Upper aerodigestive tract | 0.71 (0.33-1.54) | 1.48 (0.48-4.57) | 0.91 (0.46-1.79) | 0.09 | 0.78 |
| Urine tract | 0.93 (0.58-1.50) | 0.54 (0.16-1.81) | 0.87 (0.56-1.35) | 0.00 | 0.53 |
| Other | 1.43 (0.97-2.12) | 1.65 (0.70-3.88) | 1.39 (0.97-2.01) | 0.00 | 0.07 |

Estimates from Sweden and Denmark were derived from Cox proportional hazards models after adjustment for sex, birth year.

| Table S10. HRs with 95% CI for overall cancer and site-specific cancers among individuals with and without OCD in the sibling-matched cohorts in Sweden and Denmark | | |
| --- | --- | --- |
| Cancer types, N (%) | **Sweden** HR (95% CI)* | **Denmark** HR (95% CI)* |
| All types | 1.08 (0.97-1.20) | 0.98 (0.79-1.22) |
| Breast | 0.88 (0.66-1.17) | 0.58 (0.21-1.54) |
| Central nervous system | 1.39 (0.86-2.26) | 1.71 (0.40-7.39) |
| Cervical | 0.60 (0.26-1.42) | 0.58 (0.13-2.53) |
| Colon and rectum | 1.10 (0.78-1.56) | 1.88 (0.74-4.78) |
| Hematological | 1.21 (0.81-1.81) | 0.52 (0.17-1.60) |
| Liver and biliary passages | 2.55 (1.17-5.56) | - |
| Lung, trachea, bronchus | 1.48 (0.99-2.22) | 0.89 (0.28-2.78) |
| Melanoma | 0.75 (0.51-1.12) | 1.41 (0.67-2.98) |
| Ovarian | 2.55 (0.88-7.40) | - |
| Pancreatic | 1.06 (0.47-2.41) | - |
| Prostate | 0.90 (0.63-1.27) | 1.71 (0.40-7.33) |
| Stomach and esophagus | 1.58 (0.71-3.49) | 0.89 (0.22-3.57) |
| Testicular | 2.84 (0.99-8.11) | 0.63 (0.15-2.68) |
| Theroid and other glands | 1.70 (1.08-2.68) | 0.54 (0.12-2.43) |
| Upper aerodigestive tract | 0.71 (0.33-1.54) | 1.48 (0.48-4.57) |
| Urine tract | 0.93 (0.58-1.50) | 0.54 (0.16-1.81) |
| Other | 1.43 (0.97-2.12) | 1.65 (0.70-3.88) |

Estimates derived from marginal between-within cox regression models.
*Adjusted for sex and birth year

**Fig. S1.** Cumulative incidence functions (CIF) for overall cancer diagnosis (blue curves) and the competing risk of death prior to cancer (grey curves), comparing individuals with OCD (solid lines) to matched unexposed controls (dashed lines) in the Swedish cohort. Estimates were derived from cause-specific flexible parametric survival models (stpm2) that explicitly account for competing mortality.

**Fig. S2. Time-varying hazard ratio for incident cancer in individuals with OCD compared to unexposed individuals across follow-up.** The solid line represents the time-varying hazard ratio (HR) as a function of follow-up time, with the shaded region representing the 95% confidence interval (95 %CI). Estimates were derived from flexible parametric survival model, adjusted for sex, birth year, calendar time, and parental history of mental illness or cancer.

**Fig. S3. Standardized cumulative risk (1-survival) % and risk difference % (95% CI) between individuals with and without obsessive-compulsive disorder at age 65 for overall cancer and specific cancer types in the population-based matched cohort of Sweden.** Estimates were derived after standardization (G-formula) from flexible parametric survival models and adjusted for sex, birth year, calendar time, and parental history of mental illness or cancer.

**Fig. S4. Standardized cumulative risk (1-survival) % and risk difference % (95% CI) between individuals with and without obsessive-compulsive disorder at age 65 for overall cancer and specific cancer types in the population-based matched cohort of Denmark.** Estimates were derived after standardization (G-formula) from flexible parametric survival models and adjusted for sex, birth year, calendar time, and parental history of mental illness or cancer.
